# ADHD prevalence estimates in Italian children and adolescents: a methodological issue

**DOI:** 10.1186/s13052-018-0545-2

**Published:** 2018-09-05

**Authors:** Laura Reale, Maurizio Bonati

**Affiliations:** Laboratory for Mother and Child Health, Department of Public Health, Istituto di Ricerche Farmacologiche Mario Negri IRCCS, Via G. La Masa 19, 20156 Milan, Italy

**Keywords:** Attention deficit/hyperactivity disorder, Prevalence, Methodology, Italy

## Abstract

**Background:**

Attention deficit hyperactivity disorder (ADHD) is recognized as the most common, and most studied, developmental age disorder. Basic information, such as the most appropriate case definition and the best way to evaluate the disorder’s prevalence rate, however, remains an open issue.

**Methods:**

A comprehensive meta-analysis on the epidemiology of ADHD in Italy, which was lacking from the literature, was therefore performed to attempt to estimate the actual prevalence rate of ADHD, highlighting conceptual and quantitative differences between clinical-diagnosis and survey-based symptoms studies. The Medline, Embase, and PsycINFO databases, and the grey literature, were searched up to January 2018. The review was laid out in three main sections: an overall prevalence estimate, an epidemiological profile of ADHD symptoms, and an attempt to define the actual rate of ADHD diagnosis, as emerged from Italian studies.

**Results:**

A total of 15 unique studies were included. These contributed to estimating the prevalence of ADHD in 67,838 subjects aged 5–17, representing 9 of the 20 regions (45%) of Italy. Overall, the pooled prevalence of ADHD was 2.9% (range: 1.1–16.7%). When distinguishing studies based on case definition, however, we found an average prevalence estimate, based on symptoms criteria, of 5.9% (range: 1.4 to 16.7%) and a best-estimate prevalence rate of 1.4% (range: 1.1 to 3.1%).

**Conclusions:**

Following the case definition for epidemiological studies of ADHD, counting only subjects with an ADHD diagnosis performed and confirmed by clinical assessment would reduce the wide variability in prevalence estimates, and, above all, would both describe the real rate of subjects suffering from ADHD disorder and avoid misdiagnosis.

## Background

The exact time of onset for individual cases in psychiatry is often not known, and prevalence estimates on a given period are used as a substitute for identifying the proportion of cases of a particular disorder in a defined population [1].

Several factors can influence observed prevalence rates as the diagnostic criteria, the setting, the population studied, the type and severity of the disorder, and the comorbidities. Since measures of prevalence are also helpful in assessing health care needs and in planning health care services [2], estimates should be as accurate as possible. This is an issue especially in mental health care, where the risk of misdiagnosis and of false positives is a significant problem affecting appropriate and effective interventions, increasing the risk of medicalization and overuse of drug treatments, and creating stigma and discrimination [3, 4], as the worldwide debate also on attention deficit hyperactivity disorder (ADHD) confirms [5].

To date, ADHD is considered the most common, and most studied, developmental age disorder, even though basic information, such as the most appropriate case definition for estimating its prevalence rate, remains an open issue and leaves room for significant, debates in scientific literature [6, 7]. The reported range in prevalence is very wide (from 0.2 to 34.5%), and heterogeneity in the methodological approaches used contributes to these differences [8–12]. This is similar to the situation described for all child and mental health problems worldwide [13], supporting difficulties, greater in psychiatry than physical medicine, in discriminating disordered and non-disordered conditions [14, 15]. The 5% increase in children diagnosed as having ADHD reported in the US in recent years also suggests the need for valid tools to support diagnosis in practice [16].

ADHD is recognized as a difficult diagnosis to make accurately, not only because of the many comorbid conditions, but also for the low specificity of the core symptoms: the list of disorders or conditions that can make a child appear restless or distractible is almost endless. Making a proper diagnosis thus requires a detailed evaluation of development, educational demands, and what is expected of the child in a given circumstance and at a given time, as well as symptomatology, impairment, and risk [17, 18].

Although it is widely recognized that several ADHD symptoms, as investigated by symptom surveys or interviews, may occur as manifestations of other medical (i.e. hypoglycemia or sensory processing disorders) and psychiatric (i.e. mood, anxiety or autism spectrum disorders) disorders, few published studies have directly examined the rate and type of psychiatric and medical disorders in those previously identified as ADHD positive by teacher or parent ratings [19]. All psychiatric diagnoses are mainly clinical-based, and both subjectivity and cultural factors affect the evaluation of symptom severity (significant distress) and impairment (in social, academic or occupational functioning) in the disorder [20, 21]. Furthermore, subjects defined as having ADHD according to symptom survey-based evaluations may not truly be suffering from ADHD because they may meet only one of the five DSM-IV-TR criteria (criteria A) needed to reach a diagnosis of ADHD, and a clinical evaluation is necessary to assess the other four criteria [22]. The risk of “misdiagnosing normality” in psychiatry is high, in particular when symptom-based criteria for disorders, as in the DSM, are applied using symptom checklists, in particular in population settings and by non-medical-health professionals [14].

In such a context the goal of this study was to evaluate certain factors that can affect the prevalence of ADHD in reported studies and, consequently, the observed variability, in particular in overall pooled estimates. We analyzed the methodological approach used, and the implications in practice, of Italian ADHD studies.

## Methods

The research was approved by the Institutional Review Board of the Istituto di Ricerche Farmacologiche Mario Negri IRCCS.

### Search strategy and selection criteria

We searched the Medline, Embase, and PsycINFO databases for articles written in English and published before January 2018 using the following Medical Subject Headings and free text terms: “ADHD”, “ADD”, “attention deficit”, “attention deficit hyperactivity disorder”, “hyperkinetic disorder”, “epidemiology”, “prevalence”, “survey”, “child*”, and “adolesc*”. Studies with an Italian affiliation and point prevalence estimates of ADHD in Italy were extracted. Non indexed journals were searched for in the Google Scholar search engine by using keywords to identify potentially eligible studies. Articles written in Italian were also considered. Additionally, the reference lists of all eligible articles were scanned, as well as key Italian journals and websites, to identify additional, potentially relevant papers. Studies considered eligible were those that used the diagnostic criteria or survey instruments based on DSM-III, DSM-III-R, DSM-IV, DSM-5, or ICD-10, with samples from community, school, or clinically referred populations. We included studies with participants aged < 18 years.

### Data extraction and quality assessment

After removing duplicates, the two authors screened the titles and abstracts for adherence to eligibility criteria. In cases of uncertainty concerning eligibility, the records were discussed until a consensus was achieved. For studies deemed suitable, we obtained the full text for data extraction. References of suitable studies were searched to recover any relevant articles.

Data were extracted by the authors and involved general publication information, demographic variables of the population sample, year of sampling, setting, frame procedure, region and city, screening and diagnostic instruments used to define a case as ADHD, informant, and whether a clinical impairment evaluation was performed. The authors independently assessed each Italian study for methodological quality. The included articles were assessed by using a modified tool developed by Hoy et al. [23] for assessing risk of bias in prevalence studies that includes eight questions. These were: 1) was the study’s target population a close representation of the national population in relation to relevant variables?; 2) was the sampling frame a true or close representation of the target population?; 3) was some form of random selection used to select the sample?; 4) was the likelihood of non response bias minimal?; 5) were data collected directly from the subjects?; 6) was the study instrument that measured the parameter of interest shown to have validity and reliability?; 7) was the same mode of data collection used for all subjects?; 8) were the numerator(s) and denominator(s) for the parameter of interest appropriate? A study was considered to have a high overall risk of bias if ≤3 criteria were met, moderate risk of bias if 4 or 5 criteria were met, and low risk of bias if 6 to 8 criteria were met.

### Statistical analysis

For each retrieved study an assessment of inclusion, exclusion, and quality was performed independently by the two authors, and the inter-reviewer reliability was measured using Cohen’s Kappa statistics. Study data were analysed using Stata version 11.1 (Stata Corp, College Station, TX). Because of the differences in study sample sizes, SEs of the prevalence estimates from each study were calculated based on the exact binomial likelihood. Summary effect estimates of prevalence were calculated by using a random effects meta-regression model because there was a clear heterogeneity between the studies tested using the I^2^ statistic [24]. The study factors that might be related to prevalence estimate were first tested individually in a univariate analysis and then simultaneously in a multiple meta-regression model via likelihood ratio test conducted with R, using the ‘metafor’ package [25]. Study factors included: sample size, sampling frame, informant, quality of the study, geographical location, and diagnostic criteria. Studies were grouped according to considered factors, and the estimates were then pooled. We used the *z* test of 2 proportions to examine differences in prevalence estimates of studies by factors considered. Five studies reported prevalence estimates from different types of informants. These studies were included in univariate and multiple meta-regression analyses and in the overall pooled results for each prevalence estimate.

## Results

**Fig. 1:**
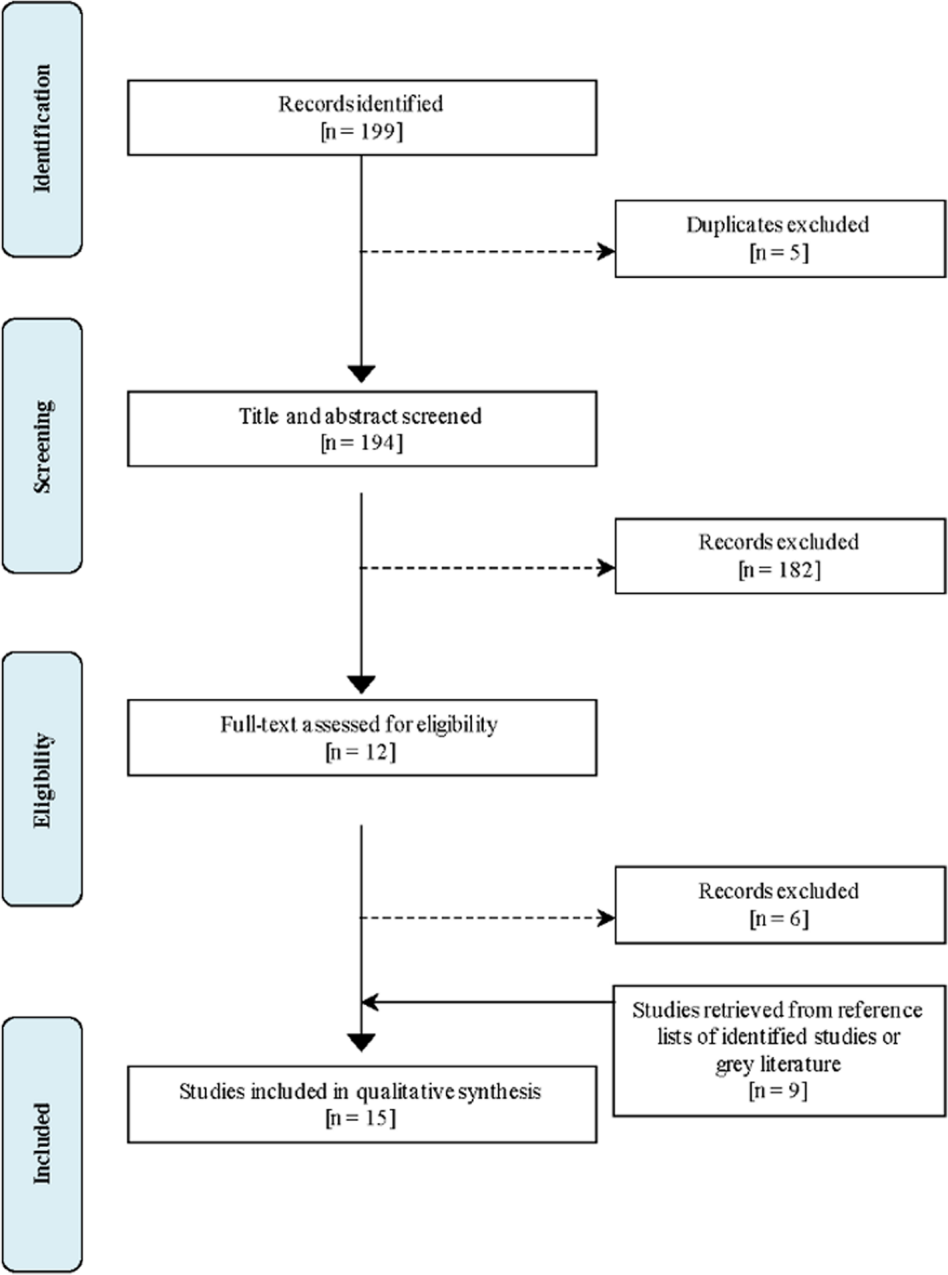
Flow diagram of Italian study retrieval and selectiongmx

Our search yielded 199 citations, 5 of which were duplicates (Fig. 1). After removal of unsuitable and ineligible studies, and the addition of 9 papers retrieved from the bibliographies of identified studies or grey literature, we had a total of 15 unique Italian studies for quality assessment and meta-analysis [26–40]. Included studies contributed 22 estimates of prevalence in 67,838 subjects, 5–17 years old, over a 30-year period (Table 1). Male sample was reported in 12 studies and lay within the range 45–55%. Both reviewers fully agreed on the choice of the pertinent studies (weighted K = 1). Studies meeting inclusion criteria were conducted in 9 of 20 Italian regions (45%) that cover 53% of the Italian 5–17 year old population. However, 7 studies (47%) were conducted in the North of the country. Among the 9 studies (60%) reporting the year of sampling the time period between the data collection and the publication ranged from 1 to 11 years (average 4). A majority of the studies was conducted in school populations (*n* = 12), while the rest were performed in clinical settings (2 in child and adolescent neuropsychiatric services and 1 in family paediatrician practices), using a whole population approach. Three of the school-based studies also involved child and adolescent neuropsychiatric services in the clinical confirmation of suspect ADHD. Overall, the methods used in the studies were rating scales, questionnaires, interviews, or other clinical tools based mainly on DSM-IV criteria (11 studies), while the remaining were conducted according to DSM-III-R (2 studies), DSM-III (1 study) and ICD-10 (1 study) criteria. The informants in the studies were teachers in 11 cases, patients in 1, and parents in 3; 2 studies included both teachers and parents.

**Table 1:** Characteristics of Italian Studies evaluated for prevalence of ADHD

First author (year of publication)	Year of data collection	Frame	Age range or mean (yrs)	Sample size	Males in the study (%)	Criteria	RoB Score	Studies using symptom-based questionnaires					Studies with clinical-based diagnosis			
								Evaluation instrument	Evaluation informant	ADHD prevalence (%)	CI 95% (binomial exact)	Males/ Females ratio	Impairment evaluation	ADHD prevalence (%)	CI 95% (binomial exact)	Males/ Females ratio
O’Leary (1985) [26]	NR	School	6–8	344	54.1	DSM-III	5	CTRS	T	12.2	8.9–16.1	6.7				
Gallucci (1993) [27]	1991	School	8–10	232	46.1	DSM-III-R	6	SQ	T	3.9	1.8–7.2	7.2				
Camerini (1996) [28]	1995	School	6–12	2557	NR	DSM-IV	2	CTRS, SQ	T	5.0	4.2–6.0	7.6				
Marzocchi (2000) [29]	1995–1996	School	7–10	973	52.0	DSM-IV	5	CTRS, SDAI, DBD	T	8.3	6.7–10.2	4.1				
Corbo (2003) [30]	1999	FP	mean: 9.5	794	52.2	DSM-IV	4	CTRS, SQ	P	2.4	1.5–3.7	NR	NR	1.5	0.8–2.6	4.8
Ciotti (2003) [31]	2003	CANPS	7–14	Population 11,980	NR	ICD-10	5						NR	1.1	0.9–1.3	3.1
Madeddu (2006) [32]	2001–2002	School	11–13	570	46.8	DSM-III-R	4	DICA-R	C	16.7	13.7–20.0	0.9	Yes	1.2	0.5–2.5	1.5
Mugnaini (2006) [33]	NR	School	6.6–7.4	1891	50.5	DSM-IV	7	Modified VADTRS	T	7.1	6.0–8.4	2.7				1.6
Zuddas (2006) [34]	NR	School	6–12	T: 1085	53.0	DSM-IV	5	DBD test	T, P	T: 8.6	7.0–10.4	1.8				
				P: 1575	55.0					P: 2.5	1.8–3.4	1.4				
										AR: 1.4	0.8–2.3	NR				
Faravelli (2009) [35]	NR	School	6–11	999	50.6	DSM-IV	5	SQ	T	5.6	4.3–7.2	2.3				
Maschietto (2012) [36]	2007–2010	CANPS	6–17	Population 24,028	NR	DSM-IV	7						Yes	1.2	1.1–1.4	1.2
Bianchini (2013) [37]	2010–2011	School	5–14	6183	51.4	DSM-IV	6	SDAI	T	7.3	6.7–8.0	3.5	Yes	3.1	2.7–3.5	5.7
Gritti (2014) [38]	NR	School	8–9	1390	45.0	DSM-IV	4	CTRS	T	2.8	2.0–3.8	0.5				
Donfrancesco (2015) [39]	2002–2003	School	7–13	1887	49.0	DSM-IV	7	SDAG, SDAI	T, P	T: 4.6	3.6–5.5	7.2	Yes	1.3	0.9–2.0	9.7
										AR: 2.2	1.6–2.3	6.3				
Zucchetti (2015) [40]	NR	School	8–10	334	48.2	DSM-IV	5	SDAI	T	10.8	7.7–14.6	1.5				

**Note**. *AR* “and rule” algorithm, *C* child, *CANPS* child and adolescent neuropsychiatric service, *CTRS* Conners’ Teacher Rating Scale, *DBD* Disruptive Behavior Disorder rating scale, *DICA-R* Diagnostic Interview for Child and Adolescent, *FP* family paediatrician, *NR* not reported, *P* parent, *SDAG* Attention and Hyperactivity Parent rating scale, *SDAI* Attention and Hyperactivity Teacher rating scale, *SQ* ad hoc study questionnaire, *T* teacher, *VADTRS* Vanderbilt Diagnostic Adhd Teacher Rating Scale

A good agreement between reviewers on the evaluation of the quality of the studies was found (weighted K = 0.61). No studies met all 8 criteria, although 93% had a low, or moderate, risk of bias. The majority of studies rated poorly for the representativeness of sample (87%).

The overall, pooled prevalence of ADHD, including all reported prevalence estimates (*n* = 22), was 4.3% (95% confidence interval [CI]:3.1 to 5.7), with a wide inter-study range of 1.1 to 16.7% (Table 1). The prevalence estimate of ADHD was, on average, 0.5% lower including, in the overall pooled analysis, only the lowest estimate of each study (3.8%, CI 2.6–5.1). In only one study of low quality the prevalence estimate of ADHD was lower for males than female [38], while in all other studies the rate for boys was 1.2–7.6 higher than for girls.

The included studies used different algorithms to estimate the number of children and adolescents with ADHD. To examine the impact of these different assessment procedures, separate prevalence estimates were calculated for each specific algorithm used. The majority of studies defined ADHD based on symptom ratings by teachers alone (8 studies). Only 2 studies required an individual to meet symptom criteria based on both parent and teacher ratings, using the “AND rule” algorithm that codes as positive only if both rates agree. Finally, 6 of 15 studies (40%) used a best estimate diagnostic algorithm in which a clinical evaluation was performed at the end of the assessment to obtain an ADHD diagnosis based on standard classification criteria (4 based on DSM-IV, 1 on DSM-III-R, 1 on ICD-10, and no study on DSM-5). Four of these 6 studies assessed a population sample and 2 a population of clinically referred subjects.

Within the univariate models, prevalence estimates for ADHD were, on average, 1.7% lower when DSM-IV criteria were used than when other criteria were used (Table 2). One study was conducted in the North, Centre, and South of Italy and prevalence estimates for ADHD were, on average, 2.2% lower compared to the North, and close to those of other geographical locations. On average, similar ADHD prevalence estimates were obtained when the informant was the clinician or both the parent and teacher (AND rule), whereas estimates were 5.1% higher when based on teacher ratings, 1% higher when based on parental reports, and 15% higher when based on child interviews. There was a significant increase in prevalence estimates when the school setting was compared with that of the population (at school estimates were, on average 3.9% higher). Prevalence estimates were, on average, 2.8% lower when study sample sizes were > 1000 participants, and 1.5% higher when the quality of the study was low or moderate (RoB score ≤ 5). Prevalence estimates were, on average, slightly lower in studies published before 2006.

**Table 2:** Association between study factors and ADHD estimates

Study factors	Univariate analyses				Multivariate analyses			
	Estimated prevalence difference %	95% CI		*P*	Estimated prevalence difference %	95% CI		*P*
		Min	Max			Min	Max	
Diagnostic criteria (DSM-IV as reference)								
Other criteria	1.65	1.23	2.07	< 0.0001	1.21	-11.53	9.11	0.7944
Geographical location (North, Center and South as reference)								
Northern Italy	2.17	1.53	2.81	< 0.0001	−1.39	-14.42	11.65	0.8123
Central Italy	−0.46	−1.15	0.23	0.1772	−8.91	-21.66	3.83	0.1454
Southern Italy	0.58	−0.11	1.27	0.1134	−5.92	-18.35	6.52	0.3045
Case definition (clinician as reference)								
AND rule	−0.12	−0.56	0.32	0.6161	−0.64	-12.21	10.93	0.9013
Parent	0.96	0.32	1.60	0.0003	2.09	−9.81	14.00	0.6957
Teacher	5.05	4.67	5.43	< 0.0001	12.88	4.28	21.48	0.0087
Child	15.14	12.08	18.20	< 0.0001	26.01	9.96	42.07	0.0057
Origin of sample (population as reference)								
School	3.87	3.60	4.14	< 0.0001	4.41	−8.59	17.41	0.4569
Family Pediatrician Practice	0.76	0.07	1.45	0.0051	7.75	-17.67	33.16	0.5022
Quality (RoB score ≥ 6 as reference)								
RoB score ≤ 5	1.45	1.14	1.76	< 0.0001	−5.34	-14.25	3.56	0.2038
Sample size (> 1000 participants as reference)								
≤ 1000 participants	2.77	2.13	3.41	< 0.0001	4.21	−5.63	14.05	0.3528
Year of study publication (≥ 2006 as reference)								
< 2006	−0.03	−0.37	0.31	0.8688	−0.04	−9.62	9.55	0.9932
				Intercept	15.44	−0.89	31.77	0.0609

According to both the clinical and methodological diversity of the retrieved studies, all univariate analyses revealed that all considered covariates were significantly associated with heterogeneity of prevalence estimates.

After entering all study factors into a multivariate meta-regression, only teacher and child case definition remained significant (Table 2).

## Discussion

Performing a clinical assessment to make a diagnosis in mental health care is necessary to define whether a subject suffers from a psychiatric disorder or not. In our opinion, therefore, selecting an appropriate case definition that is supported by a clinician-based diagnosis is a critical step to estimating the real prevalence rate of ADHD.

This opinion is not entirely new: 20 years ago, Swanson et al. [6] clearly wrote that an ADHD diagnosis should be based on clinical history since this allows one to define “the combination of inattentive, hyperactive, and impulsive behaviour as a disorder when these behaviours are severe, developmentally inappropriate, and impair function at home and school”. They continued on to say that “rating scales, with the specific ADHD symptoms, have been developed and provide a systematic approach for documenting clinical history, but these are commonly compromised by rater-specific effects and thus should be confirmed by interview”. This may be the main factor explaining the wide variation in prevalence estimates reported by the numerous individual studies and meta-analyses. Although documented, this study limitation is often ignored and thus increases the controversy over whether ADHD is overdiagnosed or underdiagnosed and the true prevalence rate of the disorder. The appraisal we performed of the meta-analyses confirms the weakness of the reported overall ADHD prevalence rates. Once again, also in our sample, the observed overlap of studies analysed in the meta-analyses seems unnecessary and may reflect a waste of efforts and an inefficiency in the process of summarizing evidence [41].

We performed a systematic evaluation of the rate of ADHD children and adolescents in Italy with the overall aim to distinguish studies estimating ADHD prevalence based only on symptom-surveys from studies providing a clinically comprehensive evaluation. We also aimed to provide an overall ADHD estimate, as done in previous reviews. The first rate we computed was the overall Italian prevalence of ADHD, found considering all types of samples with different case definition methodologies. As expected, considering Italy’s ADHD history – characterized by a predominantly psychodynamic-psychoanalytic approach [42] – the overall prevalence rate of 2.9%, ranging from 1.1 to 16.7% is lower than the worldwide estimate of 5.29% [43]. Our rate and/or range are similar to those that emerged from older review studies [44–47], while they differ more from those of the most recent studies [8, 9, 11, 12, 43, 48, 49]. These data lead to significant concern that there is inconsistent, wide variability, not only between the rates found in the original studies, but also between the findings of the reviews, suggesting the need to consider which frame, diagnosis criteria, and instruments the estimated and reported ADHD prevalence rates refer. According to other authors [6, 50], the prevalence of ADHD symptoms and the prevalence of ADHD diagnosis are rates that should be carefully differentiated from each other because they reflect two different subject populations, and they are also populations with different health care needs.

The first population (ADHD symptom rate) is the number of subjects presenting ADHD symptoms who could have an ADHD disorder or another psychiatric or medical disorder with similar clinical manifestations. This population is often recognized through symptom surveys compiled by parents or teachers, and needs a clinical evaluation to confirm whether ADHD is actually present. This population therefore represents the number of children and adolescents with behavioral symptoms of ADHD who need a specific evaluation by a specialist service/clinician.

From our findings, this population in Italy, calculated from studies with data rates based on only symptom-surveys, consists of about 439,000 subjects (5.9%, range: 1.4–16.7) among children and adolescents aged 5 to 17 years of the Italian paediatric population. This is, from a health care point of view, the population of subjects who need a psychiatric evaluation. This rate, based on symptom-surveys, differs from that found with the same methodological approach in Thomas and colleagues’ review (5.9 vs. 13%) [12], as well as from those based on parent (2.5%, range: 2.4–2.5%) and teacher ratings (6.7%, range: 4.5–10.8%), which are both lower compared to similar, previous analyses on the literature [8, 9, 47, 49]. This result could also be expected, however, taking into consideration that cultural factors, such as higher symptom tolerance, may modulate the interpretation of the child’s behaviours in parent and teacher evaluations [42, 51].

The second rate, the prevalence of ADHD diagnosis, is, according to us, the real rate of ADHD prevalence and refers to the number of patients presenting ADHD symptoms who have an ADHD diagnosis confirmed by a clinical evaluation. This population, similarly to the previous one, can be recognized through symptom surveys compiled by parents or teachers, but has an ADHD diagnosis and evaluation that confirm the presence of ADHD. This second population represents the number of children and adolescents with an ADHD diagnosis who need a specific treatment for ADHD.

From our findings, in Italy this population (ADHD diagnosis), calculated from studies including only patients with an ADHD diagnosis confirmed by clinical evaluation, consists of about 105,000 subjects (1.4%, range: 1.1–3.1) among the Italian paediatric population aged 5 to 17 years. From a health care point of view, this is the population of patients who need treatment. In line with previous comments, only a few review studies calculated the prevalence of ADHD diagnosis separately from the overall rate. It is even more important to keep in mind the distinction between diagnostic procedures if we consider that our findings (1.4%, range: 1.1–3.1) differ from those of Willcutt [9] (5.9%, range: 4.6–7.5) even when the better estimate diagnostic procedure is employed.

Finally, although we found similar rates between the overall ADHD prevalence (2.9%; range: 1.1–16.7) and the ADHD diagnosis prevalence (1.4%, range: 1.1–3.1), when diagnostic case definition based on clinical evaluation is used, comparisons of the range rates suggest that the differences between these homogenous types of studies are small, and, thus, in our opinion, more accurate.

Results should be interpreted in the context of two main limitations. First, the number of studies included is small and the methodological approach is heterogeneous so the findings that emerged may not be similar to those of other contexts or countries. Second, although this is the first study analyzing the overall ADHD prevalence in Italy, all data originate from a single country. This may affect the comparability of the reported findings and the generalization of the results to a worldwide scenario would therefore be inappropriate.

## Conclusions

Epidemiological studies concerning ADHD need more efforts to identify the cases, to assess the prevalence, and to use administrative databases as provided by the American Agency for Healthcare Research and Quality (AHRQ) [52]. In our opinion, considering only subjects with an ADHD diagnosis performed and confirmed by full clinical assessment (according to European and international guidelines) as a case definition for epidemiological ADHD studies would reduce the wide variability in ADHD estimates previously described. Above all, it would represent the real rate of subjects suffering from ADHD disorder and would avoid misdiagnosis.

Mental health is certainly a public health issue, and many disorders arise in childhood. To support the promotion of mental wellbeing and the primary prevention of psychiatric condition [53] knowledge of the true dimension of the problem – in this case the ADHD prevalence – is fundamental for planning and achieving appropriate treatments and interventions.

## Abbreviations

ADHD: Attention deficit hyperactivity disorder
AHRQ: American Agency for Healthcare Research and Quality
AR: “and rule” algorithm
CANPS: Child and Adolescent Neuropsychiatric Service
CI: Confidence interval
CTRS: Conners’ Teacher Rating Scale
DBD: Disruptive Behavior Disorder rating scale
DICA-R: Diagnostic Interview for Child and Adolescent
DSM: Statistical Manual of Mental Disorders
DSM-III: Statistical Manual of Mental Disorders. Third edition
DSM-III-R: Statistical Manual of Mental Disorders. Third edition. Revised
DSM-IV: Diagnostic and Statistical Manual of Mental disorders. Fourth edition.
DSM-IV-TR: Diagnostic and Statistical Manual of Mental disorders. Fourth edition. Text revision
FP: Family Pediatrician
ICD-10: International Statistical Classification of Diseases and Related Health Problems *10th* Revision
NR: Not reported
RoB score: Risk of Bias Assessment tool
SDAG: Attention and Hyperactivity Parent rating scale
SDAI: Attention and Hyperactivity Teacher rating scale
SEs: Standard error
SQ: Study questionnaire
VADTRS: Vanderbilt Diagnostic ADHD Teacher Rating Scale
